# Epilog: Cajal’s unique and legitimated school

**DOI:** 10.3389/fnana.2014.00058

**Published:** 2014-07-02

**Authors:** Juan Lerma, Juan A. De Carlos

**Affiliations:** ^1^Instituto de Neurociencias, Consejo Superior de Investigaciones Científicas Universidad Miguel Hernández de Elche, San Juan de AlicanteSpain; ^2^Instituto Cajal, Consejo Superior de Investigaciones Científicas, MadridSpain

**Keywords:** Cajal’s school, Spanish neuroscience, growth cone carcinogenesis, JAE, plasticity, dendritic spine, synapse

## Abstract

Santiago Ramón y Cajal is recognized as the founder of modern neuroscience, his discoveries representing the fundamental pillars of our current understanding of the nervous system. As Cajal’s career spanned a critical period in Spanish history, he witnessed strong social demands for progress in culture, education, and science. Indeed, the life of Santiago Ramón y Cajal can be considered to reflect the gradual development of Spanish science from the last third of the 19th century. Cajal promoted a national movement that had important consequences for Spanish science, mainly triggered by the creation of the “Junta para Ampliación de Estudios e Investigaciones Científicas,” an instrument he established to enrich scientific research and that was later to bear such abundant fruit. The school generated by Cajal profited from this development, through which all Cajal’s disciples received fellowships to train in laboratories across Europe. Unfortunately, the Spanish Civil War disrupted this revitalization of Spanish science and provoked the diaspora of many Spanish scientists. However, a political impulse, mostly following this spirit, was resumed in Spain during the eighties that successfully led to a renaissance in Spanish science.

Santiago Ramón y Cajal lived during difficult times in Spain, a period in which science was held in poor esteem. In that era there were only a few Spanish researchers, each of who carried out their work in quite isolated conditions. Cajal was aware of this and he tried to break out of this scientific isolation by attending as many international meetings as he could and by remaining up to date with the scientific literature, personally financing these activities (see De Carlos, 2001). The earliest and possibly the most fruitful meeting he attended was the congress of the German Anatomical Society held in Berlin in 1889, where he met the world-renowned scientist Kölliker. Talking to him and presenting his ideas on the organization of the nervous system proved to be a turning point in Cajal’s career, resulting in his introduction to the international scientific community with which Cajal remained in permanent contact thereafter. The increasing popularity of Cajal that had been initiated abroad finally reached Spain in 1900, when the International Congress of Medicine (held in Paris) awarded him the prestigious Moscow prize. Thanks to this, the Spanish Government promoted Cajal, providing him with a laboratory and an endowment to support it, thereby dramatically improving his working conditions. Cajal worked in this laboratory for 33 years (the “Laboratorio de Investigaciones Biológicas”), over which time he was able to create his own scientific school. Further international recognition of his work came later, with the award of the highly prestigious “Gold Medal of Helmholtz” (1905) and the Nobel Prize (1906).

The state of science in Spain at the time of these developments appears to be a matter of some debate. The great Spanish philosopher and humanist Ortega y Gasset wrote in a newspaper article (El Imparcial, 10–08–1908): *“There is no science in Spain. our country should not be proud of Cajal’s success but rather, it should be ashamed as it has come about by chance.”* However, there are other indications suggesting that the figure of Santiago Ramón y Cajal was not the exception that proves the rule but rather, the result, perhaps somewhat serendipitously, of the slow yet significant progress of science in Spain that commenced in the latter third of the 19th century. We cannot ignore that any scientific progress in Spain at that time was brought about by the tenacious individual will of those involved, so well incarnated by Cajal. He, like others, was able to compensate for the scarcity of resources through hard work, and this philosophy is strongly imbued in Spanish scientists who must now confront the current situation in Spain. Unlike foreign colleagues, Spanish scientists have progressed to a large extent thanks to their personal sacrifices, many times above what is humanly reasonable, a behavior bordering on stubbornness.

Cajal was aware of the limited capacity to perform science in Spain and although he initially carried out his studies in isolation, his ambition was to take advantage of his success: “*Although when I began my scientific career, both due to the force of habit and by necessity, I had to trust in the value of a solitary worker, I was always concerned with founding a school of histologists and biologists, above all once the State had entrusted me with a fine and well equipped laboratory.*”

By the last third of the 19th century, a kind of intellectual, scientific, and humanistic class had emerged in Spain, which included important personalities along with Santiago Ramón y Cajal. Indeed, the “Institución Libre de Enseñanza” (roughly translated as the “Independent Institution for Education”), was the driving force behind the cultural and social renewal of Spanish society since its conception (1876). This movement brought with it certain consequences and for instance, and in response to the intense social demand, a new Ministry of Public Instruction and Fine Arts was created in 1900. This triggered the creation in 1907 of the “Junta para Ampliación de Estudios e Investigaciones Científicas,” a board to foster scientific training and research that was chaired by Cajal. One of the main activities of this board was to sponsor the sojourns of younger investigators abroad, driven by the desire for better training, and likewise it set out to oversee and foment the building of new institutes and laboratories to host these researchers on their return. The histological school generated by Cajal profited from this structure, and all of his so-called disciples received training at top research centers in France, Germany, and England. Indeed, Cajal created a solid School of Histologists that flourished for quite a few years. Jorge Francisco Tello, Domingo Sánchez, Nicolás Achúcarro, Pio del Río-Hortega, Gonzalo R. Lafora, Fernando de Castro and Rafael Lorente de Nó, stood out among his disciples and they all made significant contributions to modern neuroscience, some of which are nowadays recognized as crucial milestones (see De Carlos and Pedraza, 2014). Unfortunately, the Spanish Civil War and the subsequent 40 year long dictatorship truncated this revitalization of Spanish neuroscience. Nevertheless, it is interesting to reflect on a similar political impulse that was appropriately resurrected in Spain during the 1980s, just after the return to democracy in Spain, giving rise to a flourishing renaissance of Spanish science that persisted for many years thereafter. Thus, although the scientific descendants of Cajal can hardly be traced, all Spanish neuroscientists feel as though they belong to Cajal’s school, independent of the discipline followed, histological or not.

## CAJAL’S MOST IMPORTANT MILESTONES

**FIGURE 1:**
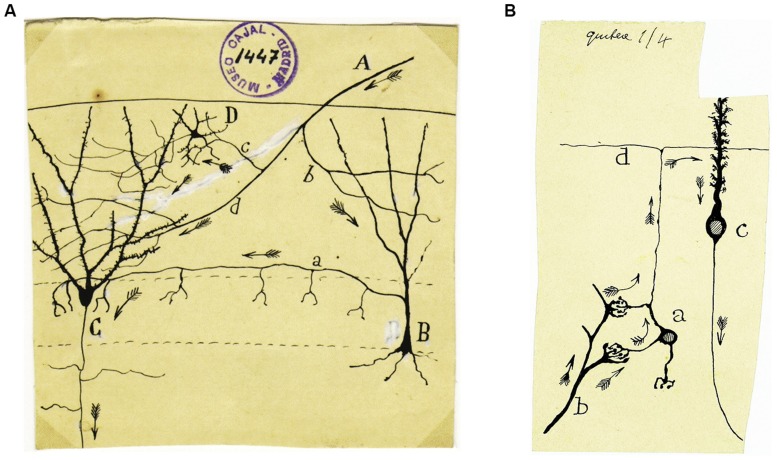
**(A) An original drawing by Cajal representing the circuit responsible for feed-forward inhibition in the dentate gyrus: A, afferent fiber; B, corpuscle of short axon terminating around the granules (i.e., basket cell); C, granule cell; D, small element of short axon.** Cajal never understood this circuit as he ignored the existence of inhibitory neurotransmission, although he did apparently speculate about the utility of this “vain loop”: “*In the figure, we show an example of the loop, apparently vain, described for afferent currents through the short axon cells*.” However, he added a few lines below: “*Not knowing the nature of the nervous movement well, it is difficult to understand how such elements increase the energy of the discharges*” (Ramón y Cajal, 1904). **(B)** In this drawing Cajal represents the cerebellar circuit in a very simple but accurate way, showing the direction of the nerve impulse with Indian arrows. Basically, from the pontine nuclei, the mossy fibers reach the cerebellum and transmit information to the granular cells. These cells conduct this information through their axons, the parallel fibers, towards the Purkinje cell dendrites, and finally, it is these cells that project the nerve impulse out of the cerebellum.

The field of neurohistology was revolutionized by Cajal’s neuronal theory and indeed, this theory provided the conceptual framework on which modern neuroscience has since been built and developed. This doctrine was the result of countless observations that Cajal made during his lifetime and his interpretation of these (Ramón y Cajal, 1954). The concept of a synapse, for instance, is fundamental to the neuron doctrine, and it was Cajal who named “nervous articulation” and provided compelling evidence for its existence. Although, it was Sherrington who coined the name, it was Cajal who initially described the functional implications of this structure, which for many was unimaginable. The prediction of information flow in the brain, as illustrated by the Indian arrows he sketched in his drawings, indicates how comprehensively Cajal understood how the nervous system functions. Indeed, Cajal’s illustrations depicted the way action currents propagate in neuronal networks. Clearly, the way in which Cajal so neatly described how information should flow in neural circuits (from axons to the dendrites or somas of other neurons) was to some extent obvious in some situations (e.g., the retina, olfactory bulb), yet it was certainly not that obvious in others (e.g., the cerebellum). Thus, Cajal not only correctly interpreted local relationships between neurons within a nucleus but also, long-range connections between nuclei (**Figure 1**). Combined with the postulate that electrical impulses propagate from dendrites to the cell body, then to the axon, Cajal could draw up what he called the *Law of Dynamic Polarization*, another fundamental contribution to neuroscience. Worth mentioning is that cortical pyramidal cells were conceptually advanced by Cajal as “psychic cells” in 1891 (Ramón y Cajal, 1891), an idea completed later on with the aid of his brother, Pedro, with whom reinforced this aspect by carrying out a comparative analysis in different species of vertebrates. As Patricia Goldman-Rakic discussed time ago (Goldman-Rakic, 2002) the name of “psychic” given by Cajal was entirely appropriate since pyramidal cells, particularly in the prefrontal cortex, process information from the outside world in the form of representation of on-going events and integrates it with previously stored knowledge, underpinning behavioral responses.

Cajal also made important advances in defining the concept of neuronal plasticity, as he claimed that areas of the brain used heavily would have richer connectivity, as their dendritic arborisation will grow with use. By contrast, he suggested that the connections in areas used less often would deteriorate and become functionally weaker. These concepts are familiar and fully accepted nowadays but amazingly, they were formulated by Cajal brain well in advance of their formal demonstration. Indeed, as pointed out by Kandel (1977), Cajal already conjectured this in 1894 on the occasion of the Croonian Lectures to the Royal Society: “…*it is possible to imagine that mental exercise facilitates a greater development of the protoplasmic apparatus and of the nervous collaterals in the part of the brain in use. In this way, pre-existing connexions between groups of cells could be reinforced by multiplication of the terminal branches of protoplasmic appendix and nervous collaterals. But the pre-existing connections could also be reinforced by the formation of new collaterals and protoplasmic expansions.”* In addition, the discovery of the dendritic spine as an anatomical and biochemically distinguishable structure was a fundamental milestone that paved the way for further studies demonstrating that this is indeed the structure where plasticity can occur, a substrate for learning and memory. Cajal was able to go further, warning that gross anatomy did not provide sufficient detail to understand mental activity. As quoted by Kandel (1977, p. 1138), Cajal wrote in 1911: “*No matter how excellent, every physiological teaching on the working of the brain based on localization leaves us ignorant of the mechanism of mental activity. These actions are certainly accompanied by molecular modifications in the nervous cells and preceded by complex changes in the relationships between neurons. To understand mental activity it is necessary to understand molecular modifications and changes in neuronal relationships. Of course one must know the complete and exact histology of cerebral centres, and their tracts, but that is not enough. It will be necessary to know the energetic transformations of the nervous system which accompany perception and thought, consciousness and emotion.”* One remains speechless upon reading these sentences, as they represent a large extent of what we currently consider to be the basis to explain learning and memory.

**FIGURE 2:**
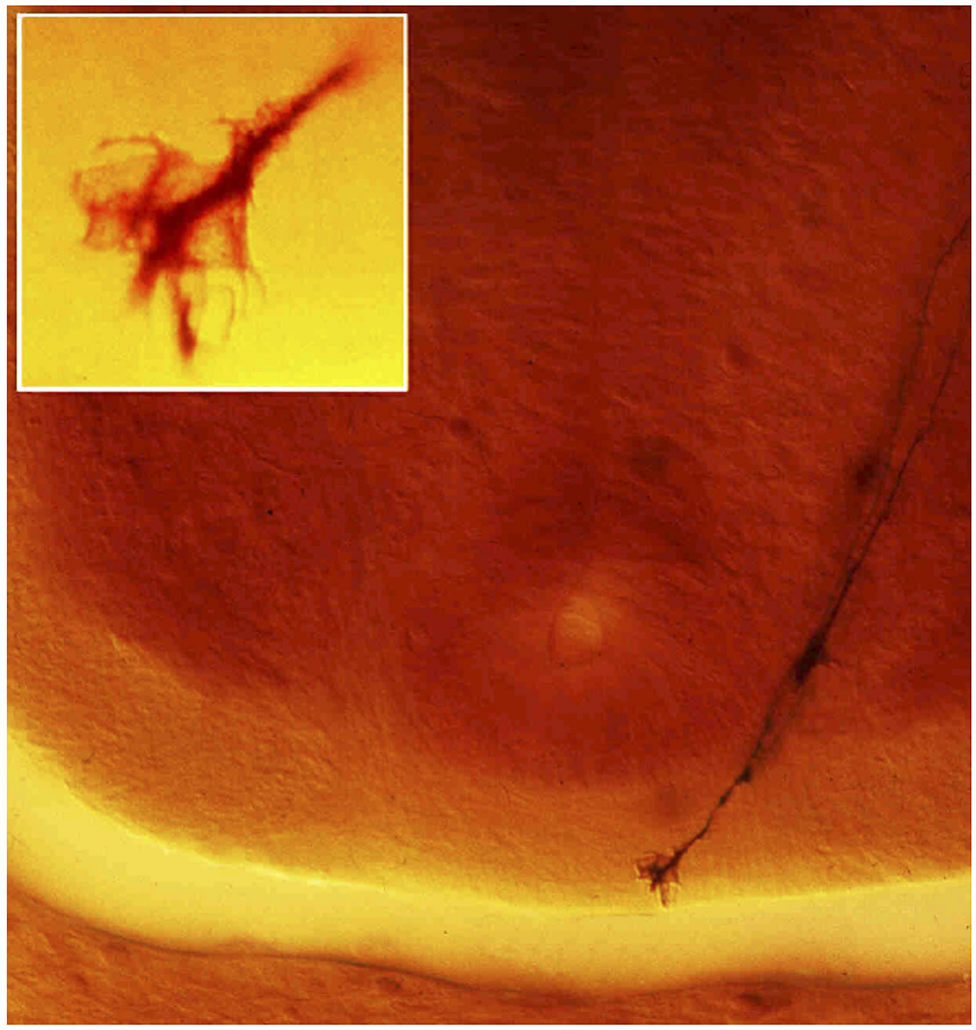
**Transverse section of the spinal cord of a day 4 Chick embryo taken by the authors from one of Cajal’s original Golgi slides (preserved at the “Instituto Cajal” in Madrid) that led him to describe the growth cone in 1890; a crucial finding in the establishment of the neuronal theory and of the trophic theory.** Cajal thought that developing neurons possessed chemotactic properties that would guide axons to their targets and he suggested that the growth cone would be guided by chemicals to find their secretory target. The figure shows two commissural axons running towards the floorplate to cross the midline. Cajal described this as follows: *“This fibre ends…in an enlargement which may be rounded and subtle, but that may also adopt a conical appearance. This latter we shall name the growth cone, that at times displays fine and short extensions…which appear to insinuate themselves between the surrounding elements, relentlessly forging a path through the interstitial matrix.*” The insert shows a higher magnification of the growth cone of one of these fibers (from Nieto, 1996).

Another indisputable milestone derived from Cajal’s work was the discovery of the growth cone (**Figure 2**). Cajal thought that during their migration, growth cones are orientated and attracted by specific chemical signals. Indeed, he used the term sniffing to illustrating how the growth cone navigates and leads the axonal fibers towards their targets. As a consequence, Cajal proposed the neurotropic theory with no more clues than a profound knowledge on how cytoarchitecture developed. As is now clear, attractive and repulsive molecules are responsible for this behavior and many such cues have now been identified, with a great deal having been determined about the signaling cascades they activate.

Cajal thoughtfully evaluated and commented publications by his contemporary colleagues and exposed his opinions on numerous aspects of science and life. Many of his thoughts and reflexions, as well as his scientific work, originally written in Spanish, have been translated into English (e.g., Ramón y Cajal, 1966; DeFelipe and Jones, 1988; Ramón y Cajal, 2002) opening paths for a general knowledge of Cajal’s work.

## IMPORTANT MILESTONES ESTABLISHED BY THE SCHOOL CREATED BY CAJAL

Cajal worked mostly alone. Perhaps his only life-long collaborator was his brother, Pedro. However, later on while having his own laboratory in Madrid, Cajal was able to create a good atmosphere in which neuroscience could progress, as can be seen if we examine some of the most representative findings of his main disciples. Cajal and Achúcarro had independent laboratories in the same building, which not only ultimately led them to collaborate but also, to share the library, instruments and technicians. Achúcarro had been trained academically abroad, occasionally visiting the clinic of Pierre Marie at the Salpêtrière, attending lectures by Babinsky and meeting neuropsychiatrists like Tanzi and Lugaro, who introduced him to the study of mental illnesses. Achúcarro had also worked in the laboratory of Alois Alzheimer, where he prepared his doctoral thesis. At that time, Cajal had just developed a new method of staining, the sublimated gold stain, that was very good to impregnate neuroglia and Achucarro had perfectioned the “Técnica de Achucarro” using tannin and ammonical oxide. In this environment and after having spent time in laboratories in Paris and Berlin, Pio del Río Hortega was accepted by Achúcarro to join his group. It was there that he was witness to the discussions between Cajal and Achúcarro (Río Hortega, 1986), who at that time insistently wanted to clarify the origin and meaning of two cellular formations close to the glial cells, which they called rod cells and granule-fatty bodies. Achúcarro died prematurely and Río Hortega took over his laboratory, from where he demonstrated the morphological characteristics of the interfascicular glia (i.e., oligodendroglia), describing this cell type as a variant of the neuroglia. He further demonstrated the mesodermal origin of the microglia, a candidate to be the third element of the nervous system. Cajal recognized these discoveries in his autobiographical book “History of my Scientific Work”: “*The discovery of microglia in the nervous centers is one of the most valuable achievements of the Spanish school”* (Ramón y Cajal, 1981). Similarly, Gonzalo Rodríguez Lafora spent time in the laboratory of Cajal as an undergraduate student and after traveling to Germany, where he studied with Theodor Ziehen, Emil Kraepelin and Alois Alzheimer in the Neurological Clinic of Munich, and to Paris where he worked with Babinski, Magnan and Dupré, he returned to Spain in 1912 to work in the “Experimental Physiology of the Nervous System Laboratory,” in the same building as Cajal’s laboratory (Moya, 1986). Remarkably, he first described progressive myoclonus epilepsy in 1911, a disease that it is currently known as Lafora’s disease (Lafora and Glueck, 1911), and that is histologically recognized by the large inclusions that accumulate inside the neuronal soma and dendrites, the so-called “Lafora bodies.” It is now known that this is an autosomal recessive disorder caused by mutations in the EPM2A or EPM2B genes that lie on human chromosome 6. EPM2A encodes a dual-specificity phosphatase called laforin, while EPM2B encodes an ubiquitin E3 ligase, called malin. These mutations provoke seizures, drop attacks, ataxia, and the development of severe dementia (e.g., Gómez-Abad et al., 2005).

Fernando de Castro began to study histology with Achúcarro but he soon entered the laboratory of Cajal, which he never abandoned. In the 1920s, Fernando de Castro started to study the sensory innervation of the aorto-carotid region, where he described, anatomically, baro-receptors (that detect pressure changes in blood vessels) and chemo-receptors (that detect changes in the chemical composition of the blood). His histological research led to the location of the carotid sinus chemo-receptors in the “glomus caroticum” and of the baro-receptors in the walls of the large arteries arising from the carotid artery (De Castro, 1926, 1928). This finding might be considered his greatest scientific contribution, since this was the very first description of a chemoreceptor. Thus, De Castro laid the anatomical basis of cardiorespiratory reflexes, leading Corneille Heymans to study the “glomus caroticum” as a center of chemosensory reflexes. Indeed, De Castro was invited by Heymans to visit his lab in Gent, where he explained his theories and the surgical approaches he used to study the glomus. From then onwards, Heymans and his collaborators reorientated their studies in an attempt to understand the physiology of the carotid body, receiving the Nobel Prize in Physiology or Medicine for this work in 1938. It is not unfair to say that Heymans’ discoveries were made possible through the studies of Fernando de Castro, which is why many members of the scientific community believed that Fernando de Castro deserved a share of the Nobel Prize awarded to Heymans.

Rafael Lorente de Nó was possibly the last direct disciple of Cajal and although he worked for the majority of his active life abroad, he did maintain a close relationship with Cajal and they frequently exchanged letters. Pedro Ramón y Cajal sent Lorente de Nó to Madrid to work in the laboratory of his brother Santiago, becoming Cajal’s youngest pupil. He spent some time at the University of Uppsala (Sweden), working with Bárány on the vestibular system, from where he moved to Berlin and after a short period in Spain, to the USA (in 1931). His fundamental research on the structure and function of the mammalian cerebral cortex and brainstem (e.g., Lorente de Nó, 1934) allowed him to make important advances in drawing up the concept of columnar organization of the cortex (Lorente de Nó, 1949), and in defining the physiology of neurons and nerve fibers (e.g., Lorente de Nó, 1947, a cornerstone of modern electrophysiology). These studies have been of tremendous importance in neuroscience, securing Lorente de Nó a prominent place in the history of this field.

## THE SIGNIFICANCE OF CAJAL’S FINDINGS IN OTHER FIELDS

**FIGURE 3:**
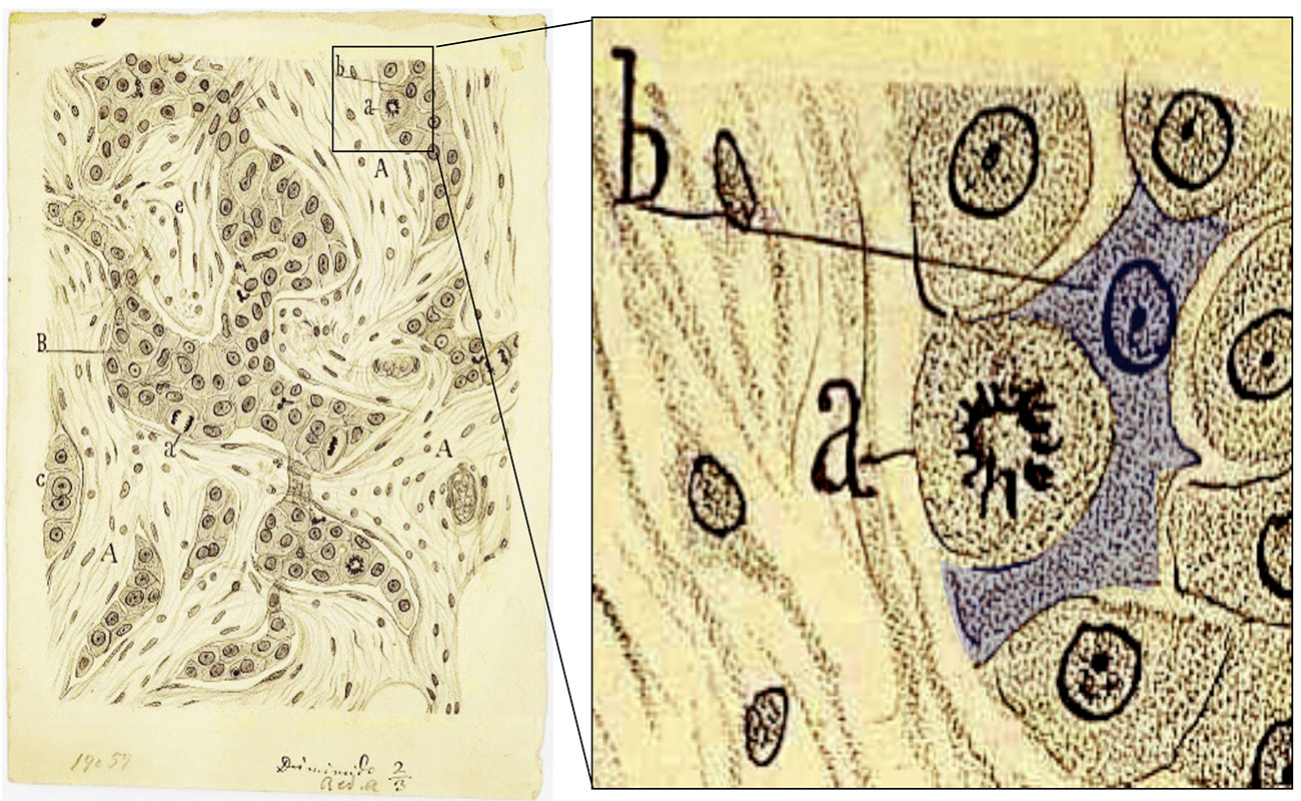
**Santiago Ramón y Cajal accurately drew and described the morphological appearance of a breast carcinoma more than 100 years ago.** The drawing on the right is adapted from Figure 48 in Ramón y Cajal (1890). The morphology of the cell highlighted as “b” (blue shadow) is believed to be the first description of the epithelial–mensenchymal transition, a first step in the metastatic cascade: “*The cells are not attached to each other… This explains their invasive ability, since free of intercellular cement, they can migrate through the connective tissue*” (from López-Novoa and Nieto, 2009, with modifications).

The remarkable ability of Cajal to derive an understanding of function from the observation of histological preparations is particularly impressive. Most neuroscientists are familiar with his descriptions on the functionality of circuits and on the role of specific types of neurons. However, many neuroscientists are unaware of the importance of his observations in other fields. In addition to his well-known book “Textura del Sistema Nervioso del Hombre y los Vertebrados,” published in Spanish in 1899 and 1904 (Volumes I and II, respectively), Cajal wrote other textbooks including the comprehensive manual of anatomic pathology (“Manual de Anatomía Patológica General”). In these, he produced all the histological slides and drawings to illustrate distinct pathological processes, in some cases with the help of his disciples, and he described the histopathology of many diseases in detail, including that of certain carcinomas (reviewed by López-Novoa and Nieto, 2009). The rational description of the cells present in mammary tumors illustrates how far ahead of his time Cajal was, even in disciplines not related to neuroscience. Indeed, he drew the invasive cells associated with breast tumors and characterized them in detail: “*The epithelial islands are not surrounded by a basement membrane…We must mention the fusiform, pear-like and star-like forms*” (Ramón y Cajal, 1890). As many oncologists recognize, it is difficult to describe the epithelial cells that acquire invasive properties in any better terms. Indeed, it also seems that Cajal was the first to describe the so-called epithelial–mensenchymal transition (EMT) and to propose its underlying mechanisms, well before this phenomenon was implicated in cancer metastasis. Effectively, Cajal’s description of breast tumors in his manual of anatomic pathology provides a premonition of this transition as the first step in the metastatic cascade (**Figure 3**). He described undifferentiated breast carcinomas as follows: “*The cells are not attached to each other…This explains their invasive ability, since free of intercellular cement, they can migrate through the connective tissue*” (Ramón y Cajal, 1890; quoted from López-Novoa and Nieto, 2009). It has since been demonstrated that what Cajal referred to as “intercellular cement” is E-cadherin, the molecule that holds epithelial cells together and that has been demonstrated to be the main target repressed to induce the EMT (Thiery et al., 2009).

## EPILOG

Cajal not only left us with a huge scientific legacy but also a sociological one, with guidelines for the way we should head into the future. Both these legacies have yielded their fruit and they have to some extent been continued. Very few aspects of his work have undergone rectification after being revisited using modern approaches. His work has not only driven our understanding of the nervous system but it also blossomed into a healthy school of neuroscientists, particularly in Spain. For this reason, many Spanish scientists can be considered to be the heirs of Cajal’s spirit, that which he imprinted on the “Junta para Ampliación de Estudios.” Many of his ideas are distilled in his words in the last chapters of his book “Reglas y Consejos para la Investigación Científica” (Guidelines and Advice for Scientific Research). In the early 1980s, his idea of sending young researchers abroad for further training was re-adopted, and many of us who took this opportunity can be considered to have been molded as Cajal’s would have wished.

## Conflict of Interest Statement

The authors declare that the research was conducted in the absence of any commercial or financial relationships that could be construed as a potential conflict of interest.
